# Microscopic and Geometric Changes in the Mandibular Condylar Head in Response to Subtle Secondary Overload: In Search of a Mechanical Origin of Condylar Hyperplasia

**DOI:** 10.3390/biology15100809

**Published:** 2026-05-20

**Authors:** Viviana Toro-Ibacache, Sonja Buvinic, Julián Balanta-Melo, Valeria Caro, Felipe Zúñiga, Ricardo Miranda-Krause, Léo Botton-Divet, John A. Nyakatura, Veronica Iturriaga, Bélgica Vásquez

**Affiliations:** 1 Faculty of Dentistry, University of Chile, Olivos 943, Independencia 8320000, Región Metropolitana, Chile; sbuvinic@u.uchile.cl (S.B.); valeriacarocotos@gmail.com (V.C.); felipe.zuniga.e@ug.uchile.cl (F.Z.); 2 Hospital Clínico San Borja Arriarán, Santa Rosa 1234, Santiago 8320000, Región Metropolitana, Chile; 3 Escuela de Odontología, Universidad del Valle, Calle 4B # 36-00, Cali 760032, Colombia; julian.balanta@correounivalle.edu.co; 4 Facultad de Medicina, Pontificia Universidad Católica de Chile, Av. Bernardo O’Higgins 340, Santiago 8320000, Región Metropolitana, Chile; rmirank@uc.cl; 5 Institut für Biologie, Humboldt Universität zu Berlin, Philipptrasse 13, Haus 2, 10115 Berlin, Germany; botton.divet.leo@gmail.com (L.B.-D.); john.nyakatura@hu-berlin.de (J.A.N.); 6 Facultad de Odontología, Universidad de La Frontera, Avenida Francisco Salazar 01145, Temuco 4780000, Región de la Araucanía, Chile; veronica.iturriaga@ufrontera.cl; 7 Department of Basic Sciences, Facultad de Medicina, Universidad de La Frontera, Avenida Francisco Salazar 01145, Temuco 4780000, Región de la Araucanía, Chile; belgica.vasquez@ufrontera.cl; 8 Center of Excellence in Morphological and Surgical Studies, Universidad de La Frontera, Avenida Francisco Salazar 01145, Temuco 4780000, Región de la Araucanía, Chile

**Keywords:** biomechanics, temporomandibular joint, condylar hyperplasia, geometric shape, microscopic structure, histology

## Abstract

In condylar hyperplasia (CH), one of the condylar processes of the mandible (CPs) grows more than its counterpart, leading to facial asymmetry. Using a mouse model of asymmetric masticatory loading, we investigated a possible mechanical origin of CH by evaluating the association between morphometric parameters of CP cartilage and bone and the mandibular load regime. Thirty-nine adult male and female mouse hemimandibles that underwent different loading regimes were used: underloaded (UL), overloaded (OL), and symmetrically loaded (SL) controls. Micro-CTs were used to assess macroscopic shape and trabecular bone (TB) parameters, and articular cartilage features were studied histologically. Data were compared using multivariate and pairwise statistics. Principal component analysis showed that OL CPs exhibit a wide range of shape variation and show thicker cartilage but display TB features indicative of ongoing remodeling. Female OL tended to show more variable and larger parameters than males. However, differences in traits between sexes and load groups were not always statistically significant. We conclude that an asymmetric increase in mandibular loading may be associated with changes in the macro-/microscopic structure of the CP, affecting males and females differently. Signs of cartilage growth and bone remodeling are found in OL individuals, which are compatible with CH features.

## Simple Summary

Condylar hyperplasia is a condition in which one side of the lower jaw grows more than the other, leading to facial asymmetry. Its causes are still not fully understood. In this study, we used a mouse model to explore whether differences in chewing forces could contribute to this uneven growth. Mice were subjected to different loading conditions: reduced load, increased load, and normal (symmetrical) load. We then examined the shape and the microscopic structure of cartilage and bone tissues. We found that increased loading on one side of the jaw was associated with changes in both overall shape and tissue structure. In particular, cartilage became thicker and bone showed signs of active remodeling. These changes were more pronounced in females than in males, although not all differences were statistically significant. Overall, our findings suggest that uneven mechanical loading during chewing may play a role in the development of condylar hyperplasia and that males and females may respond differently to these mechanical stimuli.

## 1. Introduction

Condylar hyperplasia occurs when the condylar heads of the mandible exhibit non-neoplastic, excessive growth, causing facial asymmetry when the condition presents on only one side [1]. Its diagnosis is based on clinical examination of the patient and medical imaging, including radiographs, computed tomography and single-photon emission computed tomography. Different aspects of condylar hyperplasia are of interest to researchers: diagnosis, treatment, and, particularly, the etiology, which is key to achieving prevention. However, microscopic features of unilateral condylar hyperplasia (CH) are non-specific. The head of the normal condyle is a mechanically and metabolically active structure formed by a superficial zone of dense fibrous connective tissue with collagen I parallel to the surface, a proliferative middle zone, and a deep fibrocartilaginous zone, with mainly collagen I and II [2]. The histopathological analysis of the condyles excised from CH patients shows, in most cases, a thickened deep zone, with actively growing cartilage rich in hyperplastic and hypertrophic chondrocytes, increased porosity of the subchondral cortical bone, and islands in the thickness of the subchondral trabecular bone [1,2]. This is diagnosed as active CH, and the histology is consistent with a growing condylar head [3], which is usually treated with condylar head excision. On the contrary, when there is a normal condylar histology in the enlarged condyle of an asymmetric patient, a non-active CH is confirmed. A growing condyle is normally present in growing individuals, but should not be present in adults, unless undergoing adaptive changes [4]; the CH diagnosis can be made in both young and adult individuals (more commonly in the former). Thus, the histology of active CH suggests adaptive changes in the temporomandibular joint (TMJ) based on signs of chondrogenesis and osteogenesis. This is normal in the TMJ, as its tissues derive from the cranial ectomesenchyme and have a high regeneration capacity [5]. In addition, craniomandibular muscles that generate TMJ loads have an increased capacity to adapt to environmental and mechanical requirements, producing larger or lower forces as needed [6].

Hormonal factors have also been proposed as underlying causes of CH, since CH occurs mainly in women [7]. In addition, during TMJ pain and inflammatory disorders, increased and polymorphic estrogen receptors ERα and ERβ have been found in TMJ hard tissues [7,8,9]. However, it is recognized that a hormonal factor is unlikely to be the only one, or even that a female predominance in CH may be due to women being more likely than men to seek clinical consultation when they notice facial asymmetry [7].

The hypothesis that condylar hyperplasia is an adaptation to changing TMJ mechanical conditions has been proposed but not yet tested. The articular surfaces of the TMJ in humans and other mammals, including rodents, undergo compressive forces [10,11]. Unilateral CH tends to present more frequently on the right than on the left condyle [57% vs. 43%; 7]. The right is not only the most common preferred chewing side [12], but also, when unilateral right chewing occurs with large ipsilateral muscle force, predicted loads are larger than in the balancing side’s TMJ [13]. Since hyperplastic condyles show histological features related to condylar growth, a mechanical factor is therefore a strong candidate to cause CH.

Histological analysis is the gold standard for studying tissue response to mechanical stimuli. However, it has the inherent problem of depicting a snapshot of an instant during human cartilage and bone metabolism, which could be completely different at the time of surgery, when condylectomy is performed. In addition, the mandibles of CH patients are not loaded under controlled conditions, and no control specimens are available. Single-photon emission computed tomography is an imaging method that compares radionuclide uptake between the left and right condyles; a difference >8 percentage points is considered evidence of a metabolically active condylar head. However, this method has shown different sensitivity and specificity values among studies (see examples in [14]). Three-dimensional (3D) imaging of the load-sensitive trabecular bone using micro-computed tomography (microCT), although offering a more robust way to visualize structural bone changes over time, shows the same inconvenience as histology in terms of the lack of intra-individual controls. Thus, experimental approaches combined with different analytic methods offer an opportunity to study potential causes of CH in a controlled manner. Using a multi-methodological approach that includes the analysis of macroscopic and microscopic features of the mandibular condylar structure, the present study explores a possible mechanical origin of CH using a model of asymmetric mandibular loading in adult mice. We aim to answer the question: are there microscopic and geometric features in asymmetrically overloaded condylar heads that suggest growth stimulation? The hypothesis tested is that the condyles of secondarily, functionally overloaded mandibles show geometric and histological (cartilage and bone) parameters that are more robust than those of non-overloaded (i.e., symmetrically loaded) control individuals.

## 2. Materials and Methods

Thirty-nine hemimandibles coming from 22 BALB/c adult mice (females weighing 18–20 g and males, 21–27 g) were used under the ethical approval of Universidad de Chile’s Institutional Animal Care and Use Committee (CICUA UChile #20381-ODO-UCH-e1 and #24747–ODO–UCH). Thirteen mice (8 males and 5 females, 8 weeks old), the experimental group of asymmetric masticatory loading, were subjected to a single injection of botulin toxin type A (BoNTA, 0.2 U/10 µL Onabotulinum toxin A, BOTOX^®^, AbbVie Corporation, North Chicago, IL, USA) at the right masseter muscle and of saline solution (10 μL NaCl 0.9% *w*/*v*) at the left masseter. Nine mice (6 males and 3 females) were not subject to any procedure and served as controls of normal conditions of symmetric masticatory function. After 14 days, euthanasia and dissection were undertaken, and the mandibles were placed in 10% formaldehyde buffered solution. The entire experimental procedure was detailed in Balanta-Melo et al. [15]. Adult mice were used to avoid the intrinsic augmented adaptive skeletal response of the young specimens.

Due to occasional damage, not all individuals contributed bilateral measurements. In addition, and to reduce the number of animals to comply with ethical standards, some of the animals in the control group were paired, resulting in a partially paired design. This was accounted for by including individual identity as a random effect in statistical analyses (see below), allowing appropriate handling of within-animal dependence where present. The total number of hemimandibles that were analyzed per test was subsequently uneven, maintaining a minimum sample size of five hemimandibles per group in each analysis, based on the samples reported to assess condylar head structure in previous studies [16,17]. However, due to the lack of previous studies for several of the variables studied in the present work, our results can be regarded as preliminary, providing this data that can be used for effect size and a priori sample size calculations in future studies. The sample was organized into three groups:

**Underload (UL).** The right hemimandibles of males and females, with induced paralysis by BoNTA injection. Previous studies by this research group reported, using the same animal model, that the paralyzed side shows diminished subchondral bone, which may be associated with low masticatory load [18]. These individuals are not the main focus of our research question but are used here to illustrate the effect of underload (with the subsequent overload of the contralateral side).

**Overload (OL).** The left hemimandibles of males and females with induced paralysis of the right side. In such a scenario, the side with higher muscle load typically shows more TMJ compression [19], leading to a presumed (not feasible to directly measure) secondary overload. In comparison with UL mandibles, the condylar heads on the right side have a rather normal morphology and do not show signs of degenerative changes [18] nor marked differences when their 3D structure was compared with non-intervened individuals [15]. Therefore, potential structural changes observed on the non-paralyzed side in the animal model can be considered the consequence of a secondary, subtle, or “functional” overload (i.e., not inducing degenerative changes) [20].

**Symmetric load (SL).** Left and right hemimandibles of males and females that were not subject to any procedure. Although there is no perfect anatomical nor functional symmetry in mammals, the anatomical features under normal masticatory function can be considered an appropriate baseline to study mandible asymmetry. The decision to use either side was based on a permutational multivariate analysis of variance (PERMANOVA) on trabecular bone variables (see below), which, with 10,000 rounds of permutations to calculate the *p*-value without assumptions about data distribution, showed that neither sex (F = 1.76, *p* = 0.21) nor side (F = 0.18, *p* = 0.84) affected the variance of SL data. Since right–left subtle differences are part of normal jaw development, using both sides of the SL group of the mandibles was deemed appropriate to avoid bias.

### 2.1. Data

Three variables were compared among groups: condylar head geometry, cartilage thickness and bone histomorphometry. For the first and last variables, microCT imaging was used. For cartilage thickness, classic histological processing followed by analysis under a transmitted-light microscope was used.

**Imaging.** To study bone microstructure and geometry, the dissected mandibles were imaged with a Yxlon FF20 microCT scanner at Humboldt-Universität (Berlin, Germany), using 45 kV, 110 µA, an aluminum filter of 0.5 mm, an exposure time of 3 Hz, and an isometric voxel size of 5.8 µm, at least 12 times smaller than the average mouse trabecular thickness (see Section 3. Results for details).

**Histological processing.** To study articular cartilage, classic histological analysis was performed after microCT imaging, as it is the key structure showing changes in CH cases. Mandibles were decalcified in 5% *w*/*v* buffered ethylenediaminetetraacetic acid (EDTA) for 4 days, then dehydrated in ascending concentrations of ethyl alcohol, rinsed in xylene, and paraffin-embedded orienting the major anteroposterior axis of the condylar head parallel to the bottom surface of the embedding mold. Sections of 5 µm thick were cut following the major (anteroposterior) axis of the condylar head. Then, one slice per hemimandible was selected (the most centrally located with the best quality) and stained with Safranin O and fast green according to the manufacturer’s instructions and mounted using Entellan© (Merck, Darmstadt, Germany).

### 2.2. Analyses

**Figure 1:**
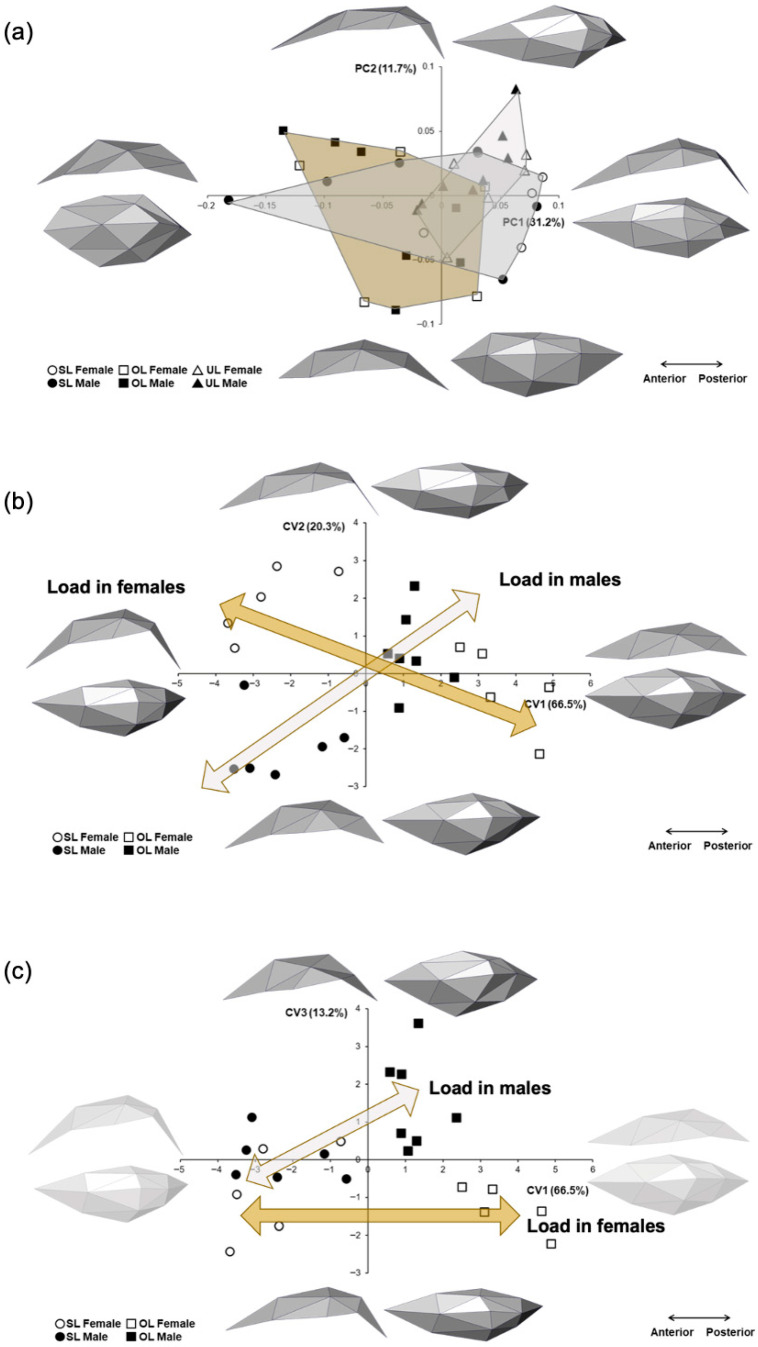
Geometric morphometric analysis. (**a**) Principal components (PC) analysis of all groups. (**b**) Canonical variate (CV) analysis of OL and SL groups, first vs. second CV and (**c**) first vs. third CV. In the PCA, the UL group shows more distinctive features, with a mediolateral narrowing and the highest point displaced towards posterior. In the CVA, the arrows depict the tendency in shape variation in females (yellow arrow) and males (light arrow), which start from very different basal (SL) shapes to converge towards a broader shape. OL = overload; SL = symmetric load; UL = underload.

**Condylar head geometry.** After segmentation of the microCT images using Avizo (v. 9.3, ThermoFischer, Waltham, MA, USA), 3D reconstructions of the condylar heads were generated. Thirteen landmarks were digitally placed on the articular surface of 36 of the condylar heads, representing the main macroscopic varying features seen in mice under the same conditions in a previous study [15]; i.e., mediolateral and anteroposterior extension, and convexity of the articular surface (see Figure 4 in [15]). Briefly, landmarks were placed on the border of the condylar head (most anterior, posterior, medial and lateral points), on the highest point on the articular surface, and then mid-distance between border landmarks and mid-distance between the highest point and the anterior, posterior, medial and lateral points (the vertices in the surface warpings; Figure 1). In addition, the centroid size, a geometric (shape-independent) size parameter, was obtained and used as an overall size reference measure in microstructure analysis.

Since the surface of the condylar head lacks type I landmarks [21] that can potentially induce significant error on the results, the landmarks were placed on a subsample of 28 condylar heads by two observers and a single observer, and then a Procrustes Analysis of Variance was performed on shape variables (i.e., 3D coordinates aligned to eliminate the effect of scale, rotation and translation) to assess the interindividual variance relative to the variance introduced by landmarking error [22] using MorphoJ (v. 1.08.02; [23]). The result showed that interindividual variance was lower than interobserver error but larger than intraobserver measurement error (F = 1.34, *p* < 0.001). Since data landmarking was supervised, these differences reflect the intrinsic difficulty of landmark placement in this anatomical region rather than random analytical inconsistency. Hence, the data from a single observer (the most experienced observer) was deemed useful for subsequent analyses. After Procrustes fit, a principal components analysis (PCA) was used to explore general shape variation among the three groups. Based on the results of the PCA where UL depicts a more unique pattern than OL/SL (see below), only the OL and SL (control) groups were compared using canonical variate analysis (CVA) to maximize the visualization of shape differences among groups. MorphoJ was used for PCA and CVA. Data was exported to Avizo and shape variation among groups was depicted with surface warping using custom-built polygon files (.ply). For statistical inference of differences in shape variation between biologically meaningful group pairs, linear mixed-effects models were fitted to principal component scores (PCs) 1 and 2 (accounting for 39.6% and 13.7% of explained variance, respectively), with sex and load condition as fixed effects and individual identity included as a random effect to account for the non-independence of left and right hemimandibles. Pairwise comparisons between groups were conducted using estimated marginal means, with *p*-values and degrees of freedom calculated using the Kenward–Roger approximation. All analyses were performed in R (version 4.6.0), using the packages geomorph, lme4, lmerTest, and emmeans.

**Cartilage thickness.** The sections of 30 condylar heads were visualized, photographed and measured by a blinded observer using the SWIFT SW380T Trinocular microscope equipped with a SWIFT 10-megapixel camera and Swift Imaging 3.0 software (Swift Microscope World, Carlsbad, CA, USA). Each slide contained anteroposterior sections where the anterior, central and posterior sections were visible and divided for measurement in three similarly sized portions from the surface. To reduce intraobserver error, cartilage thickness was measured three times, on different days by the same observer, in the thickest part of the anterior, central, and posterior portions of the cartilage, considering the inferior limit as the deepest point where the continuous, red-stained cartilage band is seen. The average value of the repeated measurements was used in subsequent analysis, after testing statistically via permutational analysis of variance (PERMANOVA; [24]) that values did not differ significantly among repetitions (F = 0.31, *p* = 0.9471). The relative importance of total cartilage thickness (measured from the articular surface) and hyperplastic/hypertrophic cartilage (HPT; measured from the most marked color change between the most superficial blue-stained cartilage and the red-stained cartilage) thickness per area was first assessed using PCA. Both PERMANOVA and PCA were performed in PAST [25]. The most relevant measurements yielded by PCA were analyzed using linear mixed-effects models, with sex and load condition as fixed effects and individual identity included as a random effect to account for paired measurements. Pairwise comparisons were conducted using estimated marginal means with appropriate correction for multiple comparisons. These tests were performed in R.

**Trabecular bone microstructure.** The sets of micro-CT images of 36 condylar heads were imported into Dragonfly software (v. 2022.2, Comet Technologies Canada Inc., Montreal, QC, Canada). Dragonfly’s Bone Analysis Wizard tools were used to define a volume of interest in each condylar head. The 3D bone structure was then segmented automatically using the Kohler method available in the software tool, double checking that the tissue was correctly selected throughout the image stack. The following parameters were analyzed: anisotropy (SVD); bone volume (BV; mm^3^); bone volume fraction (BV/TV); connectivity density (Conn.D; mm^−3^); total volume (TV; mm^3^); trabecular number (Tb.N; m^−1^); average trabecular separation (Tb.Sp; mm); and average trabecular thickness (Tb.Th; mm). In addition, centroid size, a landmark-based geometric size estimator from shape analysis, was used. The variables were first explored using correlation matrix-based PCA in PAST to assess group patterns and identify the most relevant variables, defined as those with loadings of 0.75 (either positive or negative). The most relevant variables yielded by PCA were analyzed using linear mixed-effects models, with sex and load condition as fixed effects and individual identity included as a random effect to account for paired measurements. Pairwise comparisons were conducted using estimated marginal means with appropriate correction for multiple comparisons. These tests were performed in R.

The distribution of individuals per analysis is shown in Table 1.

## 3. Results

### 3.1. Condylar Head Geometry

The PCA of Procrustes coordinates (Figure 1a) of 36 condyles shows that the SL (control) group completely spans PC1, thus depicting a wide variation range. OL individuals are less widespread in PC1 but comparatively more spread in PC2. UL individuals are found in a small area towards the positive end of PC1 and PC2, meaning that they show more distinctive features with a mediolateral narrowing and a posteriorly displaced highest point. PC1, which accounts for almost three times the variation observed in PC2, comprises individuals ranging from anteroposteriorly shorter and wider condylar heads to longer and mediolaterally narrower condylar heads. PC2 comprises individuals with more localized changes in the condylar heads: contours and elevation of the highest point of the condylar heads, which are seen in the middle portion in individuals towards the negative end of PC2 and in the posterior region in individuals towards the positive end. There is no clear separation between sexes.

In order to reduce the weight of the UL group in the subsequent analysis, the CVA results comparing only OL and SL individuals showed that in males and females, the main cause of variation (CV1) is loading and, secondarily, sex (CV2 and CV3). Interestingly, males and females change differently because they start from a different baseline (i.e., the shape of the SL individuals): males tend to have a tall condylar head that under load broadens and gets wider and slightly taller, while females change from tall to more flat and broad (Figure 1b,c). None of the biologically relevant comparisons (SL_males_ vs. SL_females_, OL_males_ vs. OL_females_, SL_males_ vs. OL_males_ and SL_females_ vs. OL_females_) yielded statistically significant differences (Table 2).

### 3.2. Cartilage Thickness

**Figure 2:**
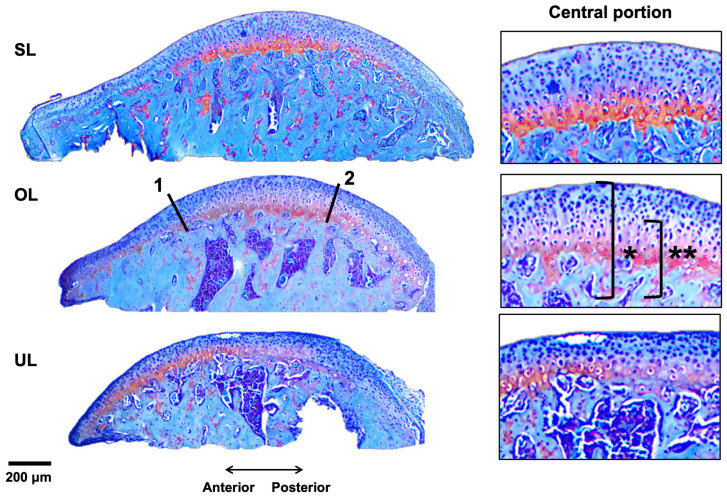
Histological analysis of condyles. **Left**: complete mediolateral view (Safranin O stain, 100X). Lines 1 and 2 mark the limits set for anterior, central and posterior portions. **Right**: close up to the central portion. OL = overload; SL = symmetric load; UL = underload; * = total cartilage; ** = HPT cartilage.

The descriptive histological analysis shows that, irrespective of sex, the OL group tends to exhibit an increased thickness of total and HPT cartilage, which is more pronounced in the central portion. The chondrocytes in this group seem comparatively more vertically aligned. In addition, the OL and UL groups show larger marrow spaces than SL mice (Figure 2). Table 3 summarizes the observed measurements.

**Figure 3:**
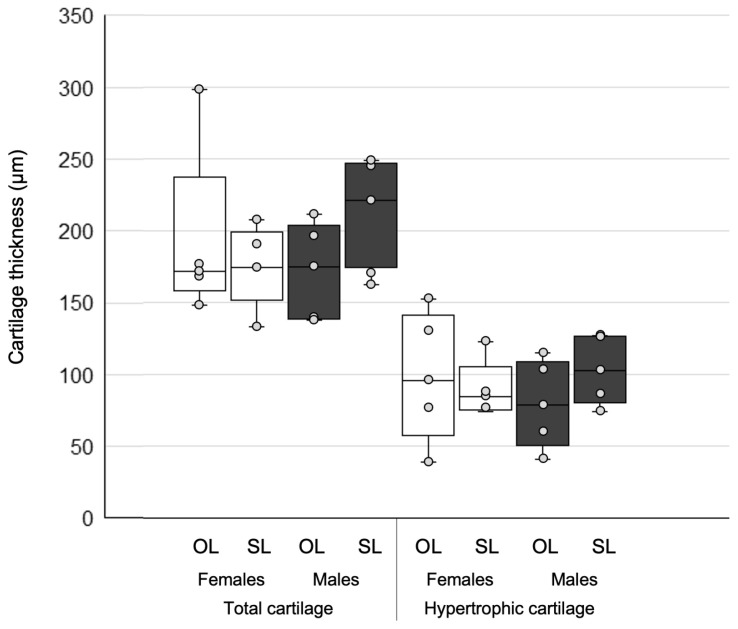
Cartilage thickness at the central portion of the condyle, both total and the HPT layer alone. OL = overload; SL = symmetric load.

The PCA showed that the first principal component (PC1) explained 64.8% of the variance. In PC1 total cartilage thickness and HPT cartilage thickness, both at the central portion, were the variables with the highest loads (0.84 and 0.5, respectively). These variables underwent subsequent pairwise statistical comparison. Results are shown in Table 4 and plotted in Figure 3.

Figure 3 shows that males and females exhibit slightly different behavior: in females, the OL group showed a comparatively larger range of thickness values, also reaching higher maximum values than OL males. SL males, on the other hand, tend to have higher thickness values than OL. In comparison and as expected, UL mice, in both males and females, have lower thickness values.

### 3.3. Microstructure

**Figure 4:**
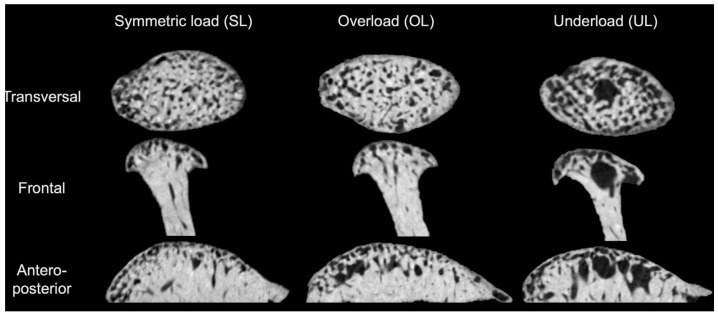
Representative microCT images of the three groups in the transversal, frontal and anteroposterior planes.

**Descriptive analysis of microCT images of condylar heads.** A qualitative assessment of the microCT images of the groups shows marked differences, mainly between UL and OL/SL. UL, as expected, shows larger medullary spaces and an overall more gracile structure when compared with OL and SL, which show similarities between them (Figure 4).

**Histomorphometric analysis of bone variables.** For the 36 condylar heads analyzed, median and range values for each of the 10 trabecular bone variables, plus centroid size, per group, are shown in Table 5. Centroid size, a parameter of geometric size derived from the condyle geometry analysis, is included here as a macroscopic size reference.

**Figure 5:**
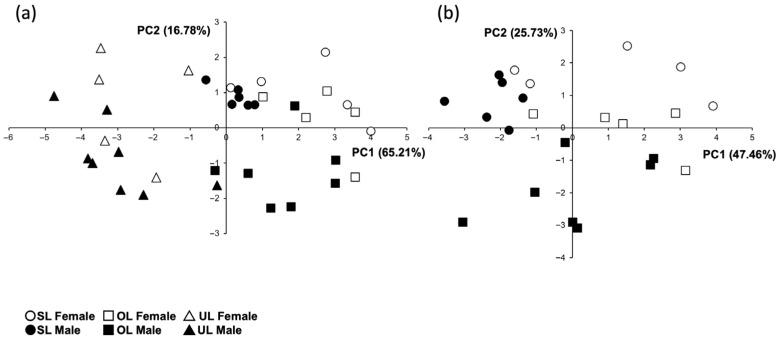
Principal components (PC) analysis of trabecular bone variables in (**a**) all the groups and in (**b**) OL and SL groups. Variance explained by each PC is shown in parentheses. OL = overload; SL = symmetric load; UL = underload.

The exploratory principal components (PC) analysis of the 10 trabecular bone variables showed that the first PC (PC1) separated UL from OL and SL (Figure 5a, all groups). Subsequently, only OL and SL (n = 23) were analyzed. In this case, the main differences (PC1) were seen between males and females and, secondarily (PC2), between load types (Figure 5b, OL and SL).

The percentage of variance explained by PCs 1 and 2 differs when analyzing OL and SL alone: PC2 is closer to the variance explained by PC1, making differences between them (sex and load) more subtle. The largest absolute loading values (at the 0.75 threshold; Table 6) were for BV, BV/TV, TV and Tb.Th in PC1 and Conn.D and Tb.N in PC2. These six variables were subsequently used for within-sex, OL/SL pairwise analysis.

**Figure 6:**
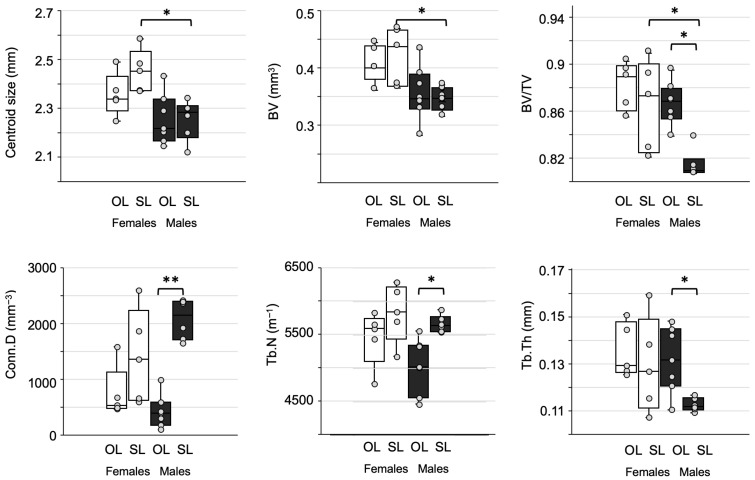
Analysis of microscopic bone variables and centroid size. Pairwise comparisons that yielded *p*-values < 0.05 are marked with asterisks. *p*-value that remained statistically significant after Bonferroni correction is shown with two asterisks. OL = overload; SL = symmetric load.

The results of the confirmatory analysis of the selected six trabecular bone variables are shown in Table 7 and Figure 6. With respect to SL, the medians of BV, Conn.D, TV and Tb.N are reduced in OL in both females and males; on the contrary, BV/TV and Tb.Th are higher in OL than in SL, in both females and males. However, the only statistically significant differences after Bonferroni correction were found in BV (males), BV/TV (males) and Conn.D (males). Interestingly, general median values of OL females (except for Tb.Th) tended to be higher than in OL males; OL females also had larger interquartile ranges. Also, the baseline values in SL females (BV, BV/TV, Conn.D, Tb.Th) showed larger variation than SL males which tend to reduce in OL females.

## 4. Discussion

Using a mouse model of asymmetric masticatory loading, this study assessed whether secondary, functional overload of the condylar head can induce structural changes similar to those observed in patients with CH. In general terms, our results showed that overloaded condyles have a comparatively larger shape variation, a tendency to have thicker articular cartilage, and trabecular bone features that suggest bone remodeling. The observed structural changes under loading showed a large interindividual variation. Therefore, more than a deterministic, causal relationship between overload and CH, our findings suggest that an increase in mechanical demands can elicit an adaptive response in TMJ tissues which are similar to features found in CH patients and that this response could be sexually dimorphic in features and magnitude. As seen in Results, several of the comparisons did not reach statistical significance. This is expected when intragroup variation is large, as it was the case here for the OL specimens. However, based on the need to assess biological and clinical significance and not only statistical significance, our discussion will evaluate trends as these are congruent with features found in CH patients.

The geometric morphometric analysis showed that the shapes of the condyles of the OL group differ slightly from SL condyles, sharing several features. A previous study by this group using the same animal model of unilateral masseter paralysis showed that the 3D geometry of the condylar head of the non-paralyzed side is similar to that of the non-treated controls (here, the symmetric load or SL group). However, their shapes are not identical, as the condylar heads of the non-paralyzed side (here, the OL group) have some comparatively more prominent features, particularly the convexity of the articular surface (Figure 4 in [15]). In the present study, when only OL and SL are compared through CVA, it can be seen that males and females change in a different way under overload: males tend to have a tall condylar head that under load broadens and gets taller and wider, while females change from tall to markedly flat (Figure 1b,c). These differences are not statistically significant; there is thus a significant interindividual variation in the morphological response of the condylar head. This is highly reflective of the adaptive capacity of the craniofacial structures [6,11].

This interindividual, sexually dimorphic variation is also seen in cartilage thickness (Figure 3) and is perhaps slightly less marked in trabecular bone parameters (Figure 6). Beyond the lack of statistical significance in comparing cartilage thickness among groups, there is a tendency toward thicker total and hyperplastic/hypertrophic (HPT) cartilage under secondary TMJ overload. The condyle cartilage, unlike the hyaline cartilage in other synovial joints, maintains throughout life a growth and remodeling capacity [4]. This capacity is tightly linked to the mechanical stimulus imposed by dental occlusion and muscle contraction during chewing [5,6]. Since the histopathological analysis of excised condyles in CH patients with active condylar growth show a persistent or thick HPT cartilage layer and no signs of tissue alteration [1,2], it can be concluded that, at least, CH and normal condyles during growth and mild overload share similar structural features. The microstructural bone changes under overload suggest ongoing remodeling changes (larger bone volume fraction, thick trabeculae, less connected), which is compatible with trabecular rearrangement under changing loading conditions. This is in agreement with previous studies showing the effect of mechanical loading on mandible microstructure and condylar bone quality [11,15,18].

Females had, in general, more variable values of cartilage and trabecular features. Although the difference with males did not show statistical significance, this tendency is relevant since CH is more prevalent in women [7]. Sexual hormones are capable of affecting bone and cartilage metabolism; males have a more robust bone metabolism in general, and women do show estrogen receptors in the TMJ, whose genetic polymorphism has been linked to a higher incidence of TMJ disorders [7,8,9].

The limited amount of time of the experimental protocol is a limitation of the present study. The animals were euthanized 14 days after overload induction, which is the time where the muscle atrophy is fully set and structural changes in the mandible are manifest [15]. However, this time might correspond to an early phase of tissue adaptation, and how the process develops over time is not known, including the possibility of systemic effects of BoNTA or even compensatory functional mechanisms. Condyle cartilage adaptation can develop for longer periods [4,11,26]. Moreover, CH itself usually develops within years in humans. Thus, a longer experimental time could add information about how tissue features evolve in time, whether they become more evident, eventually stop (with a less developed HPT cartilage layer), and whether indeed there is a sexual dimorphism in how the adaptation process develops. Based on the clinical features in CH patients, and the fact that overload scenario may be subtle, it is possible to hypothesize that once a “stable” mechanical environment is reached, condylar growth would stop. Degenerative changes, such as those seen in previous studies [11], seem less likely to occur. In addition, although reference sample sizes for TMJ structure studies in rodents were used, it was not possible to reliably calculate sample sizes a priori for our studied variables. Thus, our results can be regarded as preliminary, providing that this data can be used for effect size and a priori sample size calculations in future studies.

In spite of the limited experimental time and sample size, our results agree with those of previous studies on the effect of mechanical loads on condylar structure. Chen and colleagues [27] showed in an experimental study in developing mice under reduced masticatory forces that an increase to normal levels causes a transitory reduction in chondrocytes and collagen type II in the articular cartilage. Ravosa and colleagues [11], while studying developing rabbits fed soft and hard diets for 15 weeks, saw that the hard-diet condylar heads were larger and more mineralized, with a thicker layer of HPT cartilage on the articular surface. The same study, however, showed signs of degenerative changes in the cartilage, which the authors attributed to the extended time of the experiment. He and colleagues found similar changes using a rat model withstanding excessive magnitudes of orthopedic forces [28]. This finding is consistent with the systematic review by Betti and colleagues [20], which reported that minor changes in TMJ dynamics induce adaptive responses, whereas large changes lead to degenerative structural alterations. Since CH cannot be morphologically characterized as a pathology (there are no signs of neoplasia, and histology and imaging only show active or ceased condylar growth), our results support the idea that CH can be the result of condylar adaptation to a mechanically more demanding environment, if no evident trauma history is present. The mechanical TMJ microenvironment remains to be described in detail, which can be carried out mainly using in silico models (see, e.g., [29]). This, however, is linked to a critical problem, as results cannot be validated in vivo for ethical and technical reasons. In addition, there are reports of large inter- and intraindividual variations in the mechanical properties of the load-bearing TMJ structures, such as the disk [30] and the articular cartilage [31], which is a problem for accurate modeling of the TMJ dynamics but, more importantly, it causes different metabolic responses of the cartilage (and secondarily, of bone). This occurs because the matrix deformation will vary in magnitude and direction, thereby affecting the mechanotransduction process in chondrocytes. Although a methodological problem, this factor supports the possibility of large interindividual variations in TMJ response to asymmetric loading, which would explain why not every intrinsically asymmetric person develops CH (although in people with symptomatic TMJ disorders, condylar process asymmetry is highly prevalent; [32]).

## 5. Conclusions

Taken together, previous evidence and our current results confirm that TMJ hard tissues show signs of adaptive capacity, by remodeling and sometimes increasing their structural robusticity in response to large mechanical demands. In addition, our results highlight the relevance of sex when studying structural changes under overload, with OL females tending to increase their robusticity. It remains to be studied whether the magnitude of the adaptive response is time-dependent, and the nature of the large interindividual variations in the observed response magnitude in a larger, statistically more robust sample. However, the evidence presented here should be considered as ground knowledge in the planning of further studies hypothesizing a possible mechanical origin of CH, which can lead to decisions on its diagnosis and treatment.

## Figures and Tables

**Table 1:** Sample distribution per analysis. The data of some individuals were only suitable for some analyses and not for others. Pairing was present and accounted for during statistical analyses.

Analysis	Group	Sex	No. of Condylar Heads	No. of Individuals	No. of Paired Individuals	No. of Unpaired Individuals
Condylar head geometry	UL	M	8	8	0	8
		F	5	5	0	5
	OL	M	7	7	0	7
		F	5	5	0	5
	SL	M	6	4	2	2
		F	5	3	2	1
Cartilage thickness	UL	M	5	5	0	5
		F	5	5	0	5
	OL	M	5	5	0	5
		F	5	5	0	5
	SL	M	5	4	1	3
		F	5	4	1	3
Trabecular bone microstructure	UL	M	8	8	0	8
		F	5	5	0	5
	OL	M	7	7	0	7
		F	5	5	0	5
	SL	M	6	4	2	2
		F	5	3	2	1

UL = underload; OL = overload; SL = symmetric load; F = females; M = males.

**Table 2:** Pairwise comparisons of principal component (PC) scores obtained from linear mixed-effects models including sex and load as fixed effects and individual identity as a random effect. Estimates represent differences between group means, with associated standard errors (SE), degrees of freedom (d.f.) and *p*-values.

Comparison	PC	Estimate	SE	d.f.	*p*-Value
F: OL vs. SL	PC1	0.085	0.047	11.1	0.1
M: OL vs. SL	PC1	0.024	0.042	11.9	0.59
OL: F vs. M	PC1	−0.014	0.043	18.7	0.74
SL: F vs. M	PC1	−0.076	0.047	6.1	0.16
F: OL vs. SL	PC2	0.056	0.03	12.0	0.09
M: OL vs. SL	PC2	0.022	0.027	12.9	0.42
OL: F vs. M	PC2	−0.014	0.042	18.65	0.74
SL: F vs. M	PC2	−0.076	0.05	6.13	0.16

F = female; M = male; OL = overload; SL = symmetric load.

**Table 3:** Descriptive analysis of cartilage thickness. The reported HPT/Total Cartilage % values correspond to those observed within each group, and not to values calculated from min, max and medians.

Group	N		Total Cartilage Thickness (µm)			HPT Cartilage Thickness (µm)			HPT/Total Cartilage %		
			Anterior	Central	Posterior	Anterior	Central	Posterior	Anterior	Central	Posterior
SL_male_	5	Min	87.86	162.28	122.31	23.01	74.02	39.78	18.39	39.75	27.42
		Max	125.15	249	178.66	43.82	127.07	80.56	38.32	53.27	45.09
		Median	114.36	220.95	148.26	28.36	102.8	53.47	27.55	50.57	36.53
SL_female_	5	Min	86.54	133	127	26.22	74.13	26.66	27.56	43.48	19.76
		Max	115.53	207.65	166.75	51.11	122.8	61.63	44.24	65.92	39.09
		Median	95.15	174.39	144.76	29.98	84.55	36.14	34.31	44.34	24.93
OL_male_	5	Min	82.54	137.71	121.76	25.52	40.96	33.37	27.13	29.74	18.66
		Max	130.64	211.4	178.83	47.1	114.8	59.15	45.95	54.30	47.83
		Median	98.89	174.93	161.08	28.33	78.54	54.25	30.92	44.90	33.56
OL_female_	5	Min	95.56	148.22	117.83	35.68	38.86	44.73	25.46	26.22	31.31
		Max	152.32	298.35	155	104.66	152.5	63.43	90.12	86.39	53.83
		Median	118.99	171.79	147.08	48.52	95.77	49.09	37.34	44.49	34.62
UL_male_	5	Min	77.12	101.02	109.87	22.96	38.94	17.67	24.60	36.96	16.08
		Max	100.56	141.17	144.68	38.3	114.7	75.42	44.52	83.06	59.29
		Median	87.19	125.42	129.51	33.74	53.52	56.71	38.09	47.50	39.20
UL_female_	5	Min	86.37	108.71	82.51	14.41	39.95	27.56	16.68	31.48	27.57
		Max	120.08	154.05	146.19	61.99	83.47	52.25	57.01	54.18	35.74
		Median	108.73	126.92	113.79	40.47	58.93	33.06	41.13	50.48	33.36

OL = overload; SL = symmetric load; UL = underload; HPT = hyperplastic/hypertrophic cartilage.

**Table 4:** Pairwise comparisons of total and hypertrophic/hyperplastic (HPT) cartilage thickness at the central portion of the articular surface. The linear mixed-effects models included sex and load as fixed effects and individual identity as a random effect. Estimates represent differences between group means, with associated standard errors (SE), degrees of freedom (d.f.) and *p*-values.

Comparison	Variable	Estimate	SE	d.f.	*p*-Value
F: OL vs. SL	Total	17.4	28.5	11.7	0.55
M: OL vs. SL	Total	−40.7	28.5	11.7	0.18
OL: F vs. M	Total	20.7	26.3	15.76	0.44
SL: F vs. M	Total	−37.4	30.6	7.24	0.26
F: OL vs. SL	HPT	12.4	21.1	13.6	0.57
M: OL vs. SL	HPT	−25.2	21.1	13.6	0.26
OL: F vs. M	HPT	19.3	20.1	14.7	0.35
SL: F vs. M	HPT	−18.4	22.1	12.6	0.42

**Table 5:** Descriptive analysis of variables.

Group	Sex		Variables as Median (Range)									
		Centroid Size (mm)	Anisotropy MIL	Anisotropy SVD	BV (mm^3^)	BV/TV	Conn.D (mm^−3^)	TV (mm^3^)	Tb.N (m^−1^)	Tb.Sp (mm)	Tb.Th (mm)	Tt.Ar (mm^2^)
OL	F (n = 5)	2.34 (2.25–2.49)	0.28 (0.21–0.41)	0.55 (0.49–0.65)	0.40 (0.36–0.44)	0.89 (0.85–0.90)	530.20 (467.21–1585.72)	0.46 (0.42–0.49)	5587.27 (4762.48–5825.96)	0.05 (0.04–0.06)	0.13 (0.13–0.15)	0.21 (0.19–0.22)
	M (n = 7)	2.22 (2.15–2.43)	0.34 (0.15–0.56)	0.57 (0.52–0.61)	0.34 (0.28–0.39)	0.86 (0.84–0.90)	345.60 (100.11–987.13)	0.40 (0.34–0.44)	5166.29 (4453.92–5550.254)	0.07 (0.05–0.08)	0.13 (0.11–0.14)	0.20 (0.17–0.23)
SL	F (n = 5)	2.45 (2.37–2.58)	0.22 (0.14–0.49)	0.60 (0.54–0.63)	0.43 (0.37–0.47)	0.86 (0.82–0.91)	1468.94 (590.62–2600.80)	0.49 (0.44–0.54)	5987.62 (5169.99–6282.24)	0.04 (0.03–0.06)	0.12 (0.11–0.16)	0.22 (0.21–0.24)
	M (n = 6)	2.28 (2.12–2.34)	0.26 (0.17–0.29)	0.64 (0.60–0.72)	0.35 (0.32–0.37)	0.81 (0.81–0.84}	2148.19 (1646.18–2420.05)	0.43 (0.39–0.46)	5634.97 (5533.55–5872.62)	0.06 (0.06–0.07)	0.11 (0.11–0.12)	0.21 (0.19–0.22)
UL	F (n = 5)	2.28 (2.15–2.32)	0.38 (0.32–0.44)	0.73 (0.64–0.76)	0.25 (0.19–0.29)	0.76 (0.69–0.86)	3406.79 (1608.83–5611.98)	0.32 (0.28–0.39)	6010.19 (5213.73–6699.36)	0.08 (0.004–0.10)	0.09 (0.07–0.11)	0.15 (0.14–0.18)
	M (n = 8)	2.18 (1.89–2.18)	0.47 (0.36–0.56)	0.74 (0.67–0.81)	0.24 (0.17–0.33)	0.80 (0.72–0.84)	1823.14 (998.94–2858.16)	0.31 (0.23–0.39)	5705.44 (5179.83–6668.89)	0.08 (0.08–0.10)	0.09 (0.07–0.12)	0.15 (0.14–0.18)

UL = underload; OL = overload; SL = symmetric load; F = females; M = males; BV = bone volume; BV/TV = bone volume fraction; Conn.D = connectivity density; TV = total volume; Tb.N = trabecular number; Tb.Sp = average trabecular separation; Tb.Th = average trabecular thickness; Tt.Ar = average total (cortical + marrow) area.

**Table 6:** Loading of bone parameters in principal components (PC) 1 and 2. Those with an absolute value >0.75 are shown in bold.

Bone Parameter	PC 1 (45%)	PC 2 (30.5%)
Anisotropy (MIL)	0.04	−0.56
Anisotropy (SVD)	−0.56	0.32
**Bone volume (BV) (mm^3^)**	**0.92**	0.36
**Bone volume fraction (BV/TV)**	**0.87**	−0.39
**Connectivity density (Conn.D) (mm^−3^)**	−0.56	**0.75**
**Total volume (TV) (mm^3^)**	**0.78**	0.56
**Trabecular number (Tb.N) (m^−1^)**	−0.13	**0.88**
Average trabecular separation (Tb.Sp) (mm)	−0.73	−0.50
**Average trabecular thickness (Tb.Th) (mm)**	**0.82**	−0.48
Average total (cortical + marrow) area (Tt.Ar) (mm^2^)	0.66	0.48

**Table 7:** Pairwise comparisons of trabecular bone variables obtained from linear mixed-effects models including sex and load as fixed effects and individual identity as a random effect. Estimates represent differences between group means, with associated standard errors (SE), degrees of freedom (d.f.) and *p*-values. Statistically significant differences are shown in bold; asterisk marks the only value that remains statistically significant after Bonferroni correction.

Comparison	Variable	Estimate	SE	d.f.	*p*-Value
F: OL vs. SL	Centroid size	−0.099	0.061	11.4	0.13
M: OL vs. SL	Centroid size	0.001	0.054	12.3	0.98
OL: F vs. M	Centroid size	0.099	0.054	18.29	0.08
SL: F vs. M	Centroid size	0.2	0.06	7.11	**0.01**
F: OL vs. SL	BV	−0.021	0.03	14.7	0.49
M: OL vs. SL	BV	0.011	0.26	14.8	0.69
OL: F vs. M	BV	0.0521	0.024	15.2	0.05
SL: F vs. M	BV	0.084	0.031	14.5	**0.02**
F: OL vs. SL	BV/TV	0.014	0.016	13.4	0.4
M: OL vs. SL	BV/TV	0.053	0.014	14.0	**0.002**
OL: F vs. M	BV/TV	0.016	0.013	16.3	0.27
SL: F vs. M	BV/TV	0.054	0.017	11.9	**0.006**
F: OL vs. SL	Conn.D	−610	348	12.5	**0.1**
M: OL vs. SL	Conn.D	−1695	304	13.3	<0.0001 *
OL: F vs. M	Conn.D	325	298	17.13	0.29
SL: F vs. M	Conn.D	−760	354	9.94	0.06
F: OL vs. SL	Tb.N	−373	236	11.1	0.14
M: OL vs. SL	Tb.N	−627	210	11.9	**0.01**
OL: F vs. M	Tb.N	419	213	18.65	0.64
SL: F vs. M	Tb.N	165	234	6.13	0.51
F: OL vs. SL	Tb.Th	0.006	0.009	12.2	0.56
M: OL vs. SL	Tb.Th	0.02	0.008	13.1	**0.03**
OL: F vs. M	Tb.Th	0.004	0.008	17.42	0.64
SL: F vs. M	Tb.Th	0.018	0.009	9.26	0.09

OL = overload; SL = symmetric load; F = females; M = males; BV = bone volume; BV/TV = bone volume fraction; Conn.D = connectivity density; Tb.N = trabecular number; Tb.Th = average trabecular thickness.

## Data Availability

Data (images, datasets) can be provided upon reasonable request, and under the approval of Universidad de Chile’s Institutional Animal Care and Use Committee.

## Abbreviations

The following abbreviations are used in this manuscript:

BoNTA	Botulinum toxin type A
BV	Bone volume
CH	Condylar hyperplasia (also known as Condylar hyperactivity)
Conn.D	Connectivity density
CVA	Canonical variate analysis
EDTA	Ethylenediaminetetraacetic acid
HPT	Hyperplastic/hypertrophic (cartilage)
MIL	Mean intercept length
OL	Secondary overload
PCA	Principal components analysis
PERMANOVA	Permutational multivariate analysis of variance
SL	Symmetric load
SVD	Star volume distribution
Tb.N	Number of trabeculae
Tb.Th	Trabecular thickness
TMJ	Temporomandibular joint
Tt.Ar	Total area
TV	Total volume
UL	Underload
